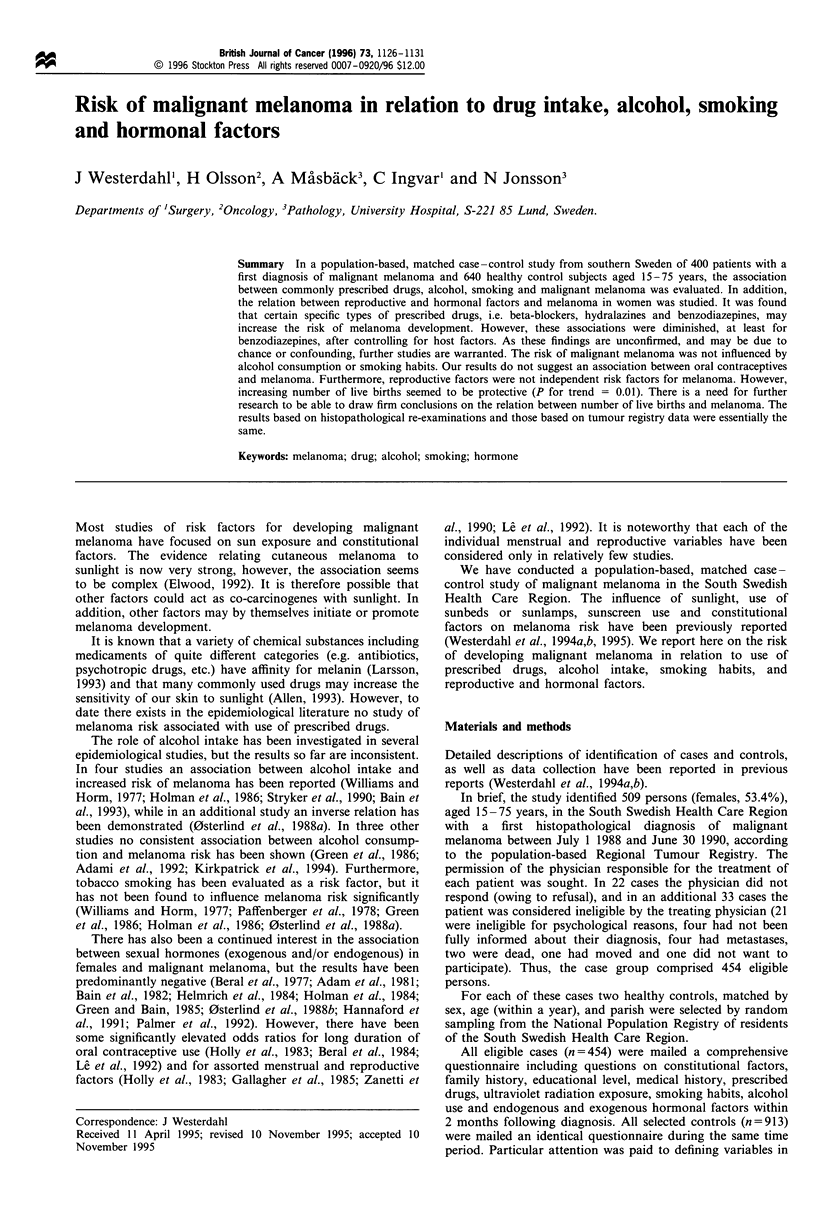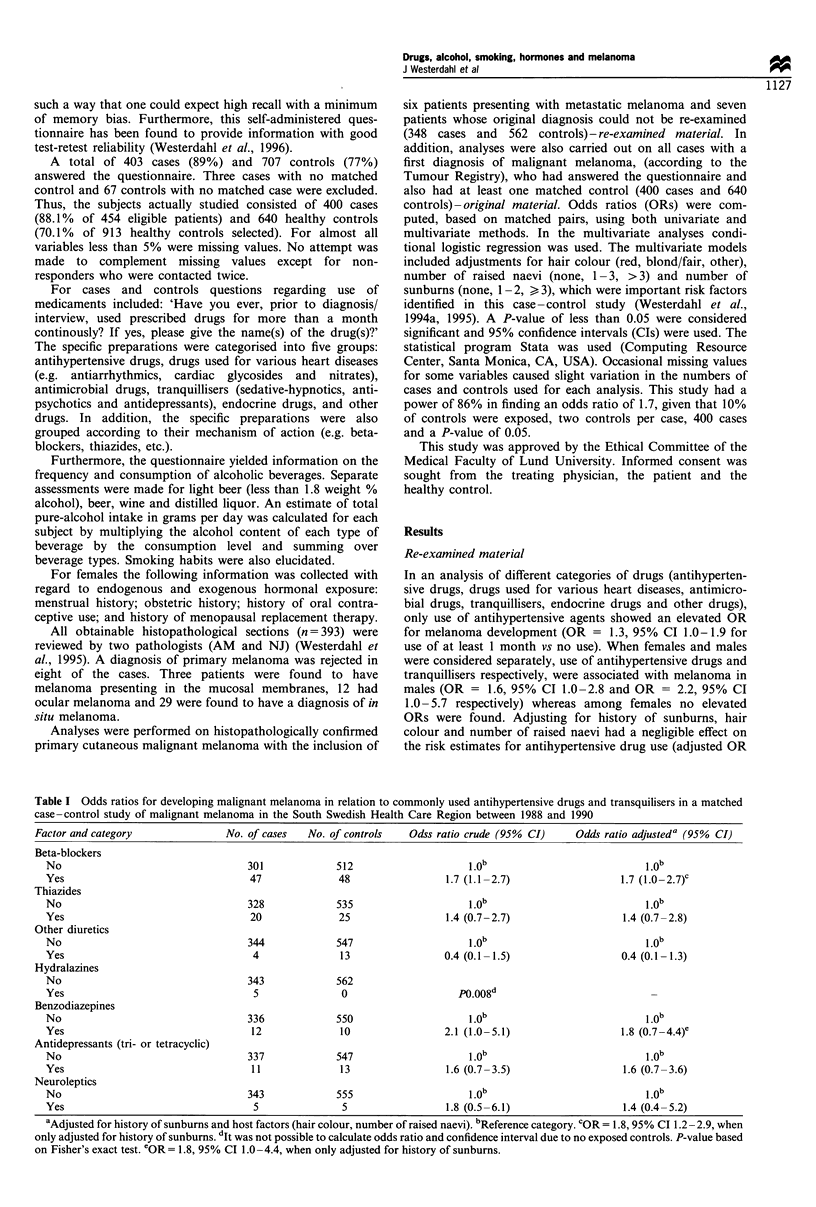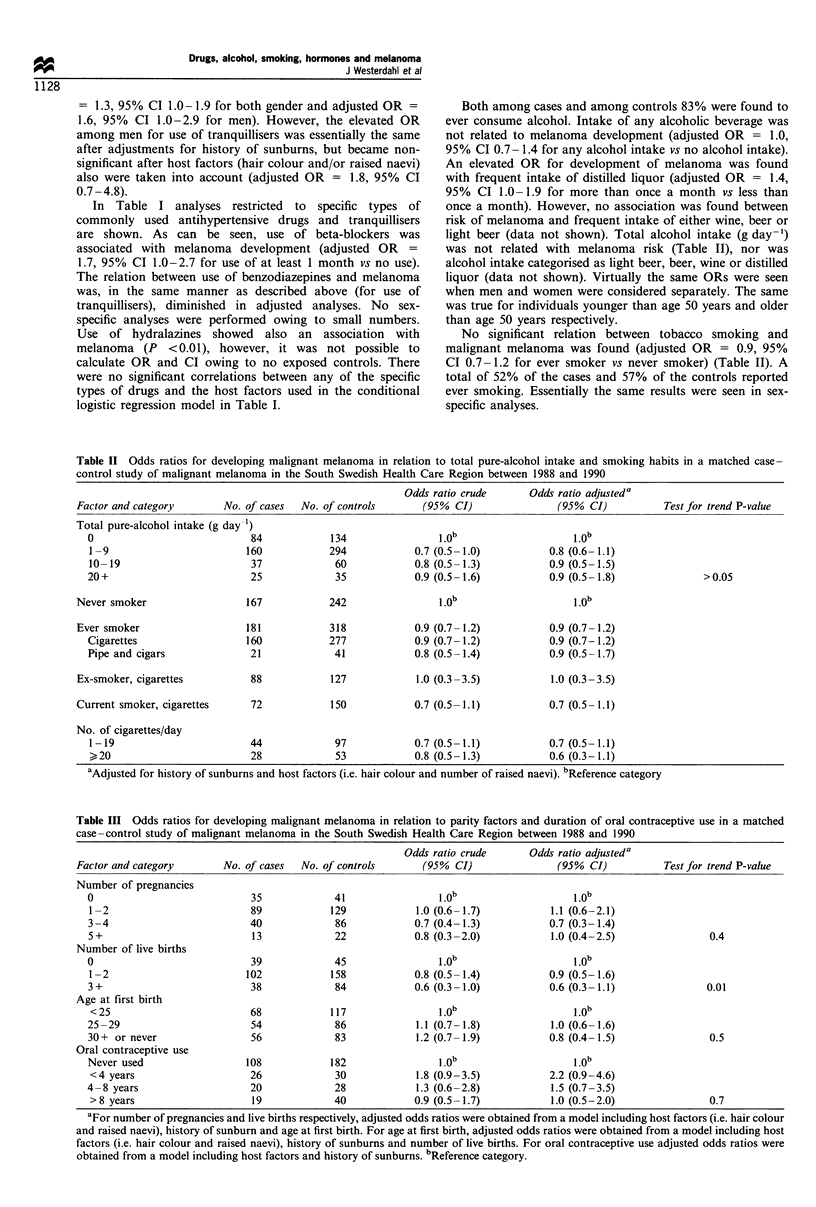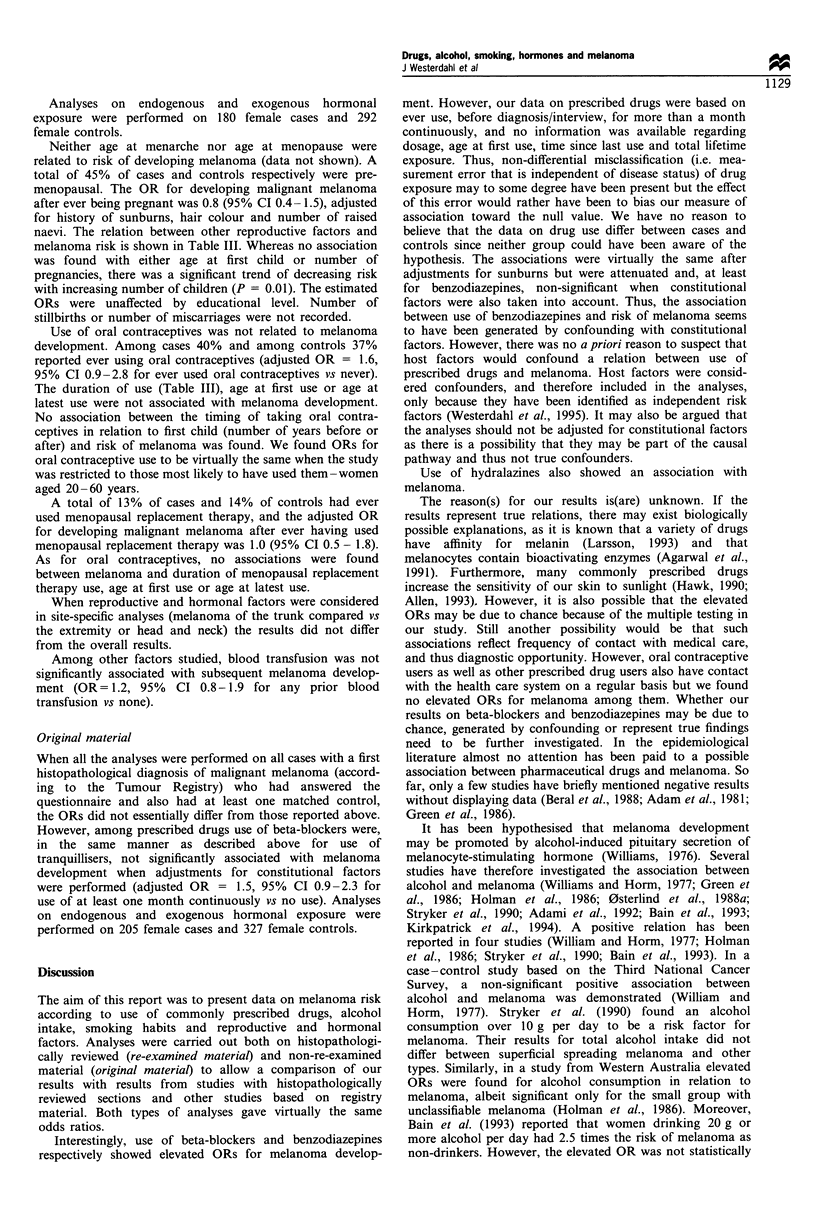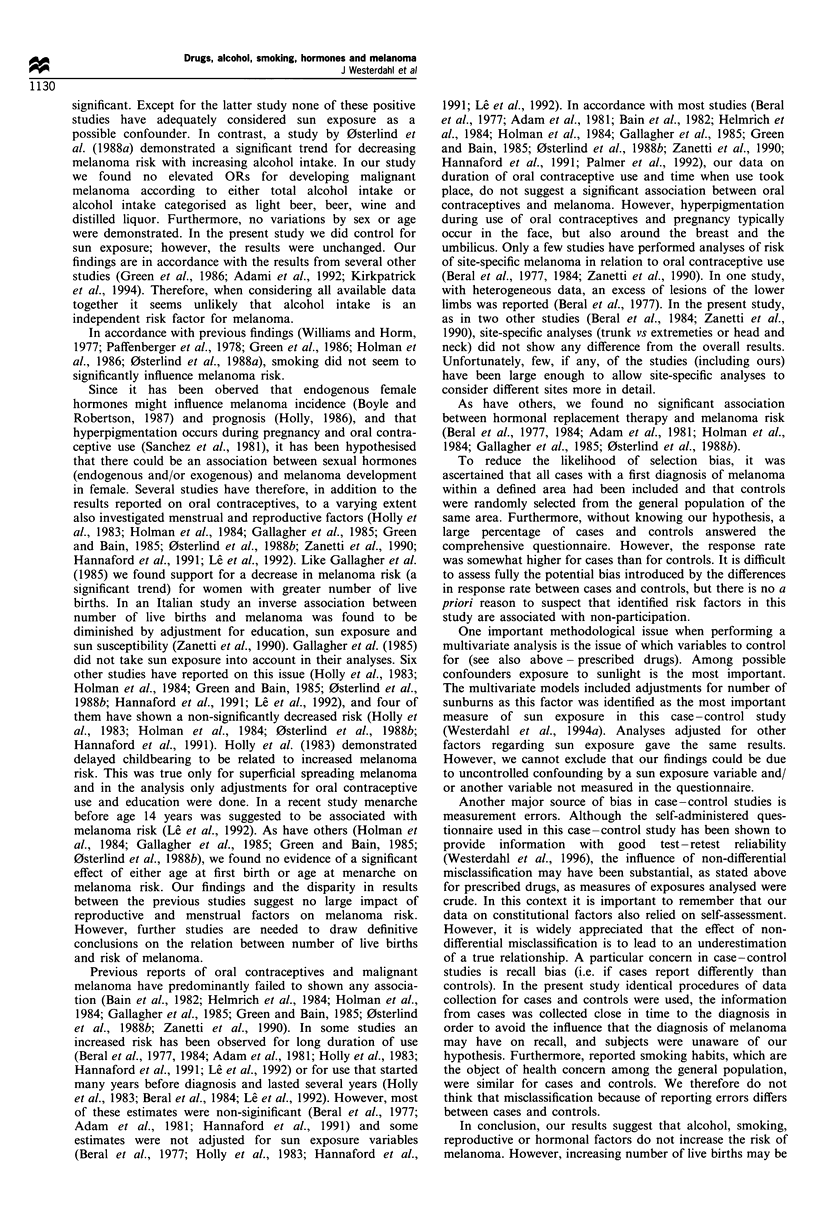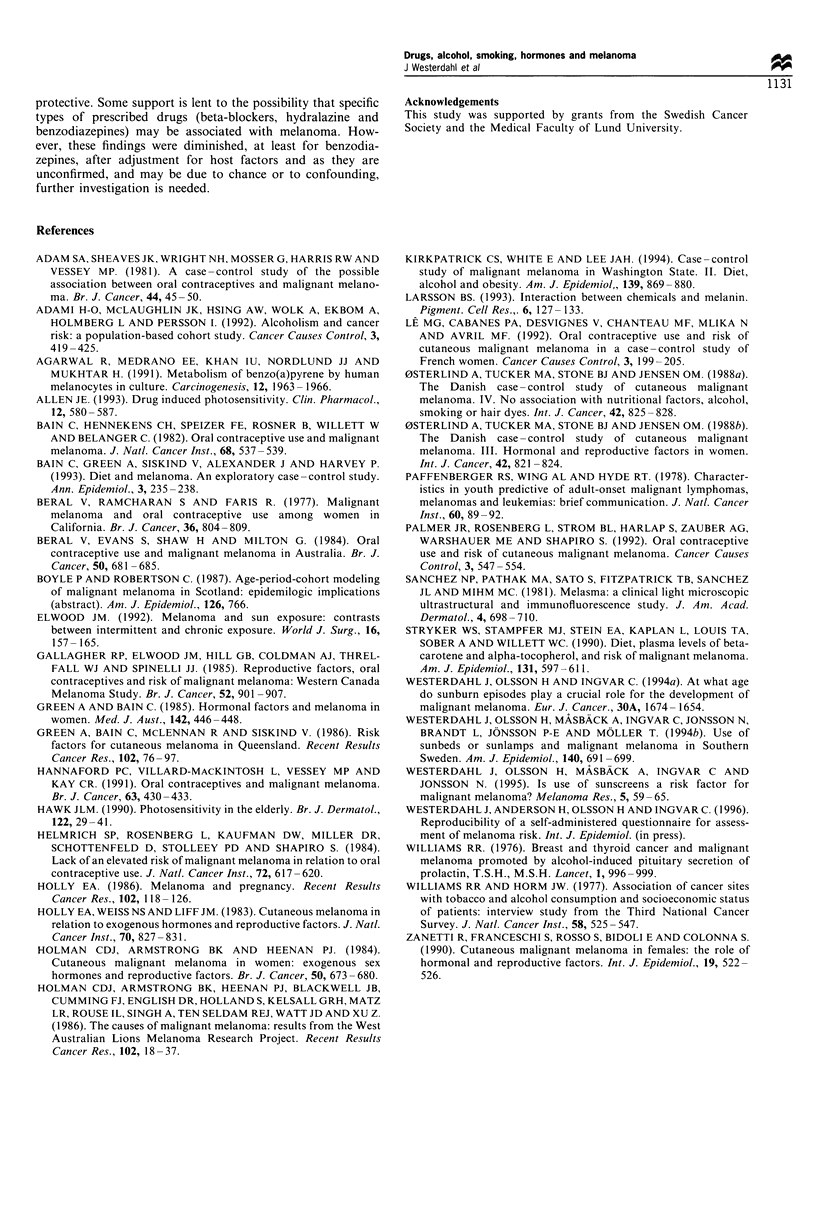# Risk of malignant melanoma in relation to drug intake, alcohol, smoking and hormonal factors.

**DOI:** 10.1038/bjc.1996.216

**Published:** 1996-05

**Authors:** J. Westerdahl, H. Olsson, A. Måsbäck, C. Ingvar, N. Jonsson

**Affiliations:** Department of Surgery, University Hospital, Lund, Sweden.

## Abstract

In a population-based, matched case-control study from southern Sweden of 400 patients with a first diagnosis of malignant melanoma and 640 healthy control subjects aged 15-75 years, the association between commonly prescribed drugs, alcohol, smoking and malignant melanoma was evaluated. In addition, the relation between reproductive and hormonal factors and melanoma in women was studied. It was found that certain specific types of prescribed drugs, i.e. beta-blockers, hydralazines and benzodiazepines, may increase the risk of melanoma development. However, these associations were diminished, at least for benzodiazepines, after controlling for host factors. As these findings are unconfirmed, and may be due to chance or confounding, further studies are warranted. The risk of malignant melanoma was not influenced by alcohol consumption or smoking habits. Our results do not suggest an association between oral contraceptives and melanoma. Furthermore, reproductive factors were not independent risk factors for melanoma. However, increasing number of live births seemed to be protective (P for trend = 0.01). There is a need for further research to be able to draw firm conclusions on the relation between number of live births and melanoma. The results based on histopathological re-examinations and those based on tumour registry data were essentially the same.


British Journal of Cancer (1996) 73, 1126-1131
© 1996 Stockton Press All rights reserved 0007-0920/96 $12.00

### Risk of malignant melanoma in relation to drug intake, alcohol, smoking and hormonal factors

J Westerdahl[1], H Olsson[2], A Måsbäck[3], C Ingvar[1] and N Jonsson[3]

*Departments of [1]Surgery, [2]Oncology, [3]Pathology, University Hospital, S-221 85 Lund, Sweden.*

**Summary** In a population-based, matched case-control study from southern Sweden of 400 patients with a first diagnosis of malignant melanoma and 640 healthy control subjects aged 15-75 years, the association between commonly prescribed drugs, alcohol, smoking and malignant melanoma was evaluated. In addition, the relation between reproductive and hormonal factors and melanoma in women was studied. It was found that certain specific types of prescribed drugs, i.e. beta-blockers, hydralazines and benzodiazepines, may increase the risk of melanoma development. However, these associations were diminished, at least for benzodiazepines, after controlling for host factors. As these findings are unconfirmed, and may be due to chance or confounding, further studies are warranted. The risk of malignant melanoma was not influenced by alcohol consumption or smoking habits. Our results do not suggest an association between oral contraceptives and melanoma. Furthermore, reproductive factors were not independent risk factors for melanoma. However, increasing number of live births seemed to be protective (*P* for trend = 0.01). There is a need for further research to be able to draw firm conclusions on the relation between number of live births and melanoma. The results based on histopathological re-examinations and those based on tumour registry data were essentially the same.

**Keywords:** melanoma; drug; alcohol; smoking; hormone

Most studies of risk factors for developing malignant melanoma have focused on sun exposure and constitutional factors. The evidence relating cutaneous melanoma to sunlight is now very strong, however, the association seems to be complex (Elwood, 1992). It is therefore possible that other factors could act as co-carcinogenes with sunlight. In addition, other factors may by themselves initiate or promote melanoma development.

It is known that a variety of chemical substances including medicaments of quite different categories (e.g. antibiotics, psychotropic drugs, etc.) have affinity for melanin (Larsson, 1993) and that many commonly used drugs may increase the sensitivity of our skin to sunlight (Allen, 1993). However, to date there exists in the epidemiological literature no study of melanoma risk associated with use of prescribed drugs.

The role of alcohol intake has been investigated in several epidemiological studies, but the results so far are inconsistent. In four studies an association between alcohol intake and increased risk of melanoma has been reported (Williams and Horm, 1977; Holman *et al.*, 1986; Stryker *et al.*, 1990; Bain *et al.*, 1993), while in an additional study an inverse relation has been demonstrated (Østerlind *et al.*, 1988*a*). In three other studies no consistent association between alcohol consumption and melanoma risk has been shown (Green *et al.*, 1986; Adami *et al.*, 1992; Kirkpatrick *et al.*, 1994). Furthermore, tobacco smoking has been evaluated as a risk factor, but it has not been found to influence melanoma risk significantly (Williams and Horm, 1977; Paffenberger *et al.*, 1978; Green *et al.*, 1986; Holman *et al.*, 1986; Østerlind *et al.*, 1988*a*).

There has also been a continued interest in the association between sexual hormones (exogenous and/or endogenous) in females and malignant melanoma, but the results have been predominantly negative (Beral *et al.*, 1977; Adam *et al.*, 1981; Bain *et al.*, 1982; Helmrich *et al.*, 1984; Holman *et al.*, 1984; Green and Bain, 1985; Østerlind *et al.*, 1988*b*; Hannaford *et al.*, 1991; Palmer *et al.*, 1992). However, there have been some significantly elevated odds ratios for long duration of oral contraceptive use (Holly *et al.*, 1983; Beral *et al.*, 1984; Lê *et al.*, 1992) and for assorted menstrual and reproductive factors (Holly *et al.*, 1983; Gallagher *et al.*, 1985; Zanetti *et al.*, 1990; Lê *et al.*, 1992). It is noteworthy that each of the individual menstrual and reproductive variables have been considered only in relatively few studies.

We have conducted a population-based, matched case-control study of malignant melanoma in the South Swedish Health Care Region. The influence of sunlight, use of sunbeds or sunlamps, sunscreen use and constitutional factors on melanoma risk have been previously reported (Westerdahl *et al.*, 1994*a*,*b*, 1995). We report here on the risk of developing malignant melanoma in relation to use of prescribed drugs, alcohol intake, smoking habits, and reproductive and hormonal factors.

### Materials and methods

Detailed descriptions of identification of cases and controls, as well as data collection have been reported in previous reports (Westerdahl *et al.*, 1994*a*,*b*).

In brief, the study identified 509 persons (females, 53.4%), aged 15-75 years, in the South Swedish Health Care Region with a first histopathological diagnosis of malignant melanoma between July 1 1988 and June 30 1990, according to the population-based Regional Tumour Registry. The permission of the physician responsible for the treatment of each patient was sought. In 22 cases the physician did not respond (owing to refusal), and in an additional 33 cases the patient was considered ineligible by the treating physician (21 were ineligible for psychological reasons, four had not been fully informed about their diagnosis, four had metastases, two were dead, one had moved and one did not want to participate). Thus, the case group comprised 454 eligible persons.

For each of these cases two healthy controls, matched by sex, age (within a year), and parish were selected by random sampling from the National Population Registry of residents of the South Swedish Health Care Region.

All eligible cases ($n=454$) were mailed a comprehensive questionnaire including questions on constitutional factors, family history, educational level, medical history, prescribed drugs, ultraviolet radiation exposure, smoking habits, alcohol use and endogenous and exogenous hormonal factors within 2 months following diagnosis. All selected controls ($n=913$) were mailed an identical questionnaire during the same time period. Particular attention was paid to defining variables in

Correspondence: J Westerdahl
Received 11 April 1995; revised 10 November 1995; accepted 10 November 1995

such a way that one could expect high recall with a minimum of memory bias. Furthermore, this self-administered questionnaire has been found to provide information with good test-retest reliability (Westerdahl *et al.*, 1996).

A total of 403 cases (89%) and 707 controls (77%) answered the questionnaire. Three cases with no matched control and 67 controls with no matched case were excluded. Thus, the subjects actually studied consisted of 400 cases (88.1% of 454 eligible patients) and 640 healthy controls (70.1% of 913 healthy controls selected). For almost all variables less than 5% were missing values. No attempt was made to complement missing values except for non-responders who were contacted twice.

For cases and controls questions regarding use of medicaments included: 'Have you ever, prior to diagnosis/ interview, used prescribed drugs for more than a month continously? If yes, please give the name(s) of the drug(s)?' The specific preparations were categorised into five groups: antihypertensive drugs, drugs used for various heart diseases (e.g. antiarrhythmics, cardiac glycosides and nitrates), antimicrobial drugs, tranquillisers (sedative-hypnotics, antipsychotics and antidepressants), endocrine drugs, and other drugs. In addition, the specific preparations were also grouped according to their mechanism of action (e.g. beta-blockers, thiazides, etc.).

Furthermore, the questionnaire yielded information on the frequency and consumption of alcoholic beverages. Separate assessments were made for light beer (less than 1.8 weight % alcohol), beer, wine and distilled liquor. An estimate of total pure-alcohol intake in grams per day was calculated for each subject by multiplying the alcohol content of each type of beverage by the consumption level and summing over beverage types. Smoking habits were also elucidated.

For females the following information was collected with regard to endogenous and exogenous hormonal exposure: menstrual history; obstetric history; history of oral contraceptive use; and history of menopausal replacement therapy.

All obtainable histopathological sections ($n = 393$) were reviewed by two pathologists (AM and NJ) (Westerdahl *et al.*, 1995). A diagnosis of primary melanoma was rejected in eight of the cases. Three patients were found to have melanoma presenting in the mucosal membranes, 12 had ocular melanoma and 29 were found to have a diagnosis of *in situ* melanoma.

Analyses were performed on histopathologically confirmed primary cutaneous malignant melanoma with the inclusion of six patients presenting with metastatic melanoma and seven patients whose original diagnosis could not be re-examined (348 cases and 562 controls) – *re-examined material.* In addition, analyses were also carried out on all cases with a first diagnosis of malignant melanoma, (according to the Tumour Registry), who had answered the questionnaire and also had at least one matched control (400 cases and 640 controls) – *original material.* Odds ratios (ORs) were computed, based on matched pairs, using both univariate and multivariate methods. In the multivariate analyses conditional logistic regression was used. The multivariate models included adjustments for hair colour (red, blond/fair, other), number of raised naevi (none, 1–3, >3) and number of sunburns (none, 1–2, ⩾3), which were important risk factors identified in this case–control study (Westerdahl *et al.*, 1994a, 1995). A *P*-value of less than 0.05 were considered significant and 95% confidence intervals (CIs) were used. The statistical program Stata was used (Computing Resource Center, Santa Monica, CA, USA). Occasional missing values for some variables caused slight variation in the numbers of cases and controls used for each analysis. This study had a power of 86% in finding an odds ratio of 1.7, given that 10% of controls were exposed, two controls per case, 400 cases and a *P*-value of 0.05.

This study was approved by the Ethical Committee of the Medical Faculty of Lund University. Informed consent was sought from the treating physician, the patient and the healthy control.

### Results

### *Re-examined material*

In an analysis of different categories of drugs (antihypertensive drugs, drugs used for various heart diseases, antimicrobial drugs, tranquillisers, endocrine drugs and other drugs), only use of antihypertensive agents showed an elevated OR for melanoma development (OR = 1.3, 95% CI 1.0–1.9 for use of at least 1 month *vs* no use). When females and males were considered separately, use of antihypertensive drugs and tranquillisers respectively, were associated with melanoma in males (OR = 1.6, 95% CI 1.0–2.8 and OR = 2.2, 95% CI 1.0–5.7 respectively) whereas among females no elevated ORs were found. Adjusting for history of sunburns, hair colour and number of raised naevi had a negligible effect on the risk estimates for antihypertensive drug use (adjusted OR

**Table I** Odds ratios for developing malignant melanoma in relation to commonly used antihypertensive drugs and transquilisers in a matched case–control study of malignant melanoma in the South Swedish Health Care Region between 1988 and 1990

| *Factor and category* | *No. of cases* | *No. of controls* | *Odss ratio crude (95% CI)* | *Odds ratio adjusted[a] (95% CI)* |
|---|---|---|---|---|
| Beta-blockers | | | | |
| No | 301 | 512 | 1.0[b] | 1.0[b] |
| Yes | 47 | 48 | 1.7 (1.1–2.7) | 1.7 (1.0–2.7)[c] |
| Thiazides | | | | |
| No | 328 | 535 | 1.0[b] | 1.0[b] |
| Yes | 20 | 25 | 1.4 (0.7–2.7) | 1.4 (0.7–2.8) |
| Other diuretics | | | | |
| No | 344 | 547 | 1.0[b] | 1.0[b] |
| Yes | 4 | 13 | 0.4 (0.1–1.5) | 0.4 (0.1–1.3) |
| Hydralazines | | | | |
| No | 343 | 562 | | |
| Yes | 5 | 0 | *P*0.008[d] | – |
| Benzodiazepines | | | | |
| No | 336 | 550 | 1.0[b] | 1.0[b] |
| Yes | 12 | 10 | 2.1 (1.0–5.1) | 1.8 (0.7–4.4)[e] |
| Antidepressants (tri- or tetracyclic) | | | | |
| No | 337 | 547 | 1.0[b] | 1.0[b] |
| Yes | 11 | 13 | 1.6 (0.7–3.5) | 1.6 (0.7–3.6) |
| Neuroleptics | | | | |
| No | 343 | 555 | 1.0[b] | 1.0[b] |
| Yes | 5 | 5 | 1.8 (0.5–6.1) | 1.4 (0.4–5.2) |

[a]Adjusted for history of sunburns and host factors (hair colour, number of raised naevi). [b]Reference category. [c]OR = 1.8, 95% CI 1.2–2.9, when only adjusted for history of sunburns. [d]It was not possible to calculate odds ratio and confidence interval due to no exposed controls. *P*-value based on Fisher's exact test. [e]OR = 1.8, 95% CI 1.0–4.4, when only adjusted for history of sunburns.

= 1.3, 95% CI 1.0–1.9 for both gender and adjusted OR = 1.6, 95% CI 1.0–2.9 for men). However, the elevated OR among men for use of tranquillisers was essentially the same after adjustments for history of sunburns, but became non-significant after host factors (hair colour and/or raised naevi) also were taken into account (adjusted OR = 1.8, 95% CI 0.7–4.8).

In Table I analyses restricted to specific types of commonly used antihypertensive drugs and tranquillisers are shown. As can be seen, use of beta-blockers was associated with melanoma development (adjusted OR = 1.7, 95% CI 1.0–2.7 for use of at least 1 month *vs* no use). The relation between use of benzodiazepines and melanoma was, in the same manner as described above (for use of tranquillisers), diminished in adjusted analyses. No sex-specific analyses were performed owing to small numbers. Use of hydralazines showed also an association with melanoma ($P < 0.01$), however, it was not possible to calculate OR and CI owing to no exposed controls. There were no significant correlations between any of the specific types of drugs and the host factors used in the conditional logistic regression model in Table I.

Both among cases and among controls 83% were found to ever consume alcohol. Intake of any alcoholic beverage was not related to melanoma development (adjusted OR = 1.0, 95% CI 0.7–1.4 for any alcohol intake *vs* no alcohol intake). An elevated OR for development of melanoma was found with frequent intake of distilled liquor (adjusted OR = 1.4, 95% CI 1.0–1.9 for more than once a month *vs* less than once a month). However, no association was found between risk of melanoma and frequent intake of either wine, beer or light beer (data not shown). Total alcohol intake (g day$^{-1}$) was not related with melanoma risk (Table II), nor was alcohol intake categorised as light beer, beer, wine or distilled liquor (data not shown). Virtually the same ORs were seen when men and women were considered separately. The same was true for individuals younger than age 50 years and older than age 50 years respectively.

No significant relation between tobacco smoking and malignant melanoma was found (adjusted OR = 0.9, 95% CI 0.7–1.2 for ever smoker *vs* never smoker) (Table II). A total of 52% of the cases and 57% of the controls reported ever smoking. Essentially the same results were seen in sex-specific analyses.

**Table II** Odds ratios for developing malignant melanoma in relation to total pure-alcohol intake and smoking habits in a matched case–control study of malignant melanoma in the South Swedish Health Care Region between 1988 and 1990

| *Factor and category* | *No. of cases* | *No. of controls* | *Odds ratio crude (95% CI)* | *Odds ratio adjusted*[a] *(95% CI)* | *Test for trend* P-*value* |
|---|---|---|---|---|---|
| Total pure-alcohol intake (g day$^{-1}$) | | | | | |
| 0 | 84 | 134 | 1.0[b] | 1.0[b] | |
| 1–9 | 160 | 294 | 0.7 (0.5–1.0) | 0.8 (0.6–1.1) | |
| 10–19 | 37 | 60 | 0.8 (0.5–1.3) | 0.9 (0.5–1.5) | |
| 20+ | 25 | 35 | 0.9 (0.5–1.6) | 0.9 (0.5–1.8) | >0.05 |
| Never smoker | 167 | 242 | 1.0[b] | 1.0[b] | |
| Ever smoker | 181 | 318 | 0.9 (0.7–1.2) | 0.9 (0.7–1.2) | |
| Cigarettes | 160 | 277 | 0.9 (0.7–1.2) | 0.9 (0.7–1.2) | |
| Pipe and cigars | 21 | 41 | 0.8 (0.5–1.4) | 0.9 (0.5–1.7) | |
| Ex-smoker, cigarettes | 88 | 127 | 1.0 (0.3–3.5) | 1.0 (0.3–3.5) | |
| Current smoker, cigarettes | 72 | 150 | 0.7 (0.5–1.1) | 0.7 (0.5–1.1) | |
| No. of cigarettes/day | | | | | |
| 1–19 | 44 | 97 | 0.7 (0.5–1.1) | 0.7 (0.5–1.1) | |
| ⩾20 | 28 | 53 | 0.8 (0.5–1.3) | 0.6 (0.3–1.1) | |

[a]Adjusted for history of sunburns and host factors (i.e. hair colour and number of raised naevi). [b]Reference category

**Table III** Odds ratios for developing malignant melanoma in relation to parity factors and duration of oral contraceptive use in a matched case–control study of malignant melanoma in the South Swedish Health Care Region between 1988 and 1990

| *Factor and category* | *No. of cases* | *No. of controls* | *Odds ratio crude (95% CI)* | *Odds ratio adjusted*[a] *(95% CI)* | *Test for trend* P-*value* |
|---|---|---|---|---|---|
| Number of pregnancies | | | | | |
| 0 | 35 | 41 | 1.0[b] | 1.0[b] | |
| 1–2 | 89 | 129 | 1.0 (0.6–1.7) | 1.1 (0.6–2.1) | |
| 3–4 | 40 | 86 | 0.7 (0.4–1.3) | 0.7 (0.3–1.4) | |
| 5+ | 13 | 22 | 0.8 (0.3–2.0) | 1.0 (0.4–2.5) | 0.4 |
| Number of live births | | | | | |
| 0 | 39 | 45 | 1.0[b] | 1.0[b] | |
| 1–2 | 102 | 158 | 0.8 (0.5–1.4) | 0.9 (0.5–1.6) | |
| 3+ | 38 | 84 | 0.6 (0.3–1.0) | 0.6 (0.3–1.1) | 0.01 |
| Age at first birth | | | | | |
| <25 | 68 | 117 | 1.0[b] | 1.0[b] | |
| 25–29 | 54 | 86 | 1.1 (0.7–1.8) | 1.0 (0.6–1.6) | |
| 30+ or never | 56 | 83 | 1.2 (0.7–1.9) | 0.8 (0.4–1.5) | 0.5 |
| Oral contraceptive use | | | | | |
| Never used | 108 | 182 | 1.0[b] | 1.0[b] | |
| <4 years | 26 | 30 | 1.8 (0.9–3.5) | 2.2 (0.9–4.6) | |
| 4–8 years | 20 | 28 | 1.3 (0.6–2.8) | 1.5 (0.7–3.5) | |
| >8 years | 19 | 40 | 0.9 (0.5–1.7) | 1.0 (0.5–2.0) | 0.7 |

[a]For number of pregnancies and live births respectively, adjusted odds ratios were obtained from a model including host factors (i.e. hair colour and raised naevi), history of sunburn and age at first birth. For age at first birth, adjusted odds ratios were obtained from a model including host factors (i.e. hair colour and raised naevi), history of sunburns and number of live births. For oral contraceptive use adjusted odds ratios were obtained from a model including host factors and history of sunburns. [b]Reference category.

Analyses on endogenous and exogenous hormonal exposure were performed on 180 female cases and 292 female controls.

Neither age at menarche nor age at menopause were related to risk of developing melanoma (data not shown). A total of 45% of cases and controls respectively were premenopausal. The OR for developing malignant melanoma after ever being pregnant was 0.8 (95% CI 0.4–1.5), adjusted for history of sunburns, hair colour and number of raised naevi. The relation between other reproductive factors and melanoma risk is shown in Table III. Whereas no association was found with either age at first child or number of pregnancies, there was a significant trend of decreasing risk with increasing number of children ($P$ = 0.01). The estimated ORs were unaffected by educational level. Number of stillbirths or number of miscarriages were not recorded.

Use of oral contraceptives was not related to melanoma development. Among cases 40% and among controls 37% reported ever using oral contraceptives (adjusted OR = 1.6, 95% CI 0.9–2.8 for ever used oral contraceptives *vs* never). The duration of use (Table III), age at first use or age at latest use were not associated with melanoma development. No association between the timing of taking oral contraceptives in relation to first child (number of years before or after) and risk of melanoma was found. We found ORs for oral contraceptive use to be virtually the same when the study was restricted to those most likely to have used them–women aged 20–60 years.

A total of 13% of cases and 14% of controls had ever used menopausal replacement therapy, and the adjusted OR for developing malignant melanoma after ever having used menopausal replacement therapy was 1.0 (95% CI 0.5 – 1.8). As for oral contraceptives, no associations were found between melanoma and duration of menopausal replacement therapy use, age at first use or age at latest use.

When reproductive and hormonal factors were considered in site-specific analyses (melanoma of the trunk compared *vs* the extremity or head and neck) the results did not differ from the overall results.

Among other factors studied, blood transfusion was not significantly associated with subsequent melanoma development (OR = 1.2, 95% CI 0.8–1.9 for any prior blood transfusion *vs* none).

### *Original material*

When all the analyses were performed on all cases with a first histopathological diagnosis of malignant melanoma (according to the Tumour Registry) who had answered the questionnaire and also had at least one matched control, the ORs did not essentially differ from those reported above. However, among prescribed drugs use of beta-blockers were, in the same manner as described above for use of tranquillisers, not significantly associated with melanoma development when adjustments for constitutional factors were performed (adjusted OR = 1.5, 95% CI 0.9–2.3 for use of at least one month continuously *vs* no use). Analyses on endogenous and exogenous hormonal exposure were performed on 205 female cases and 327 female controls.

### Discussion

The aim of this report was to present data on melanoma risk according to use of commonly prescribed drugs, alcohol intake, smoking habits and reproductive and hormonal factors. Analyses were carried out both on histopathologically reviewed (*re-examined material*) and non-re-examined material (*original material*) to allow a comparison of our results with results from studies with histopathologically reviewed sections and other studies based on registry material. Both types of analyses gave virtually the same odds ratios.

Interestingly, use of beta-blockers and benzodiazepines respectively showed elevated ORs for melanoma development. However, our data on prescribed drugs were based on ever use, before diagnosis/interview, for more than a month continuously, and no information was available regarding dosage, age at first use, time since last use and total lifetime exposure. Thus, non-differential misclassification (i.e. measurement error that is independent of disease status) of drug exposure may to some degree have been present but the effect of this error would rather have been to bias our measure of association toward the null value. We have no reason to believe that the data on drug use differ between cases and controls since neither group could have been aware of the hypothesis. The associations were virtually the same after adjustments for sunburns but were attenuated and, at least for benzodiazepines, non-significant when constitutional factors were also taken into account. Thus, the association between use of benzodiazepines and risk of melanoma seems to have been generated by confounding with constitutional factors. However, there was no *a priori* reason to suspect that host factors would confound a relation between use of prescribed drugs and melanoma. Host factors were considered confounders, and therefore included in the analyses, only because they have been identified as independent risk factors (Westerdahl *et al.*, 1995). It may also be argued that the analyses should not be adjusted for constitutional factors as there is a possibility that they may be part of the causal pathway and thus not true confounders.

Use of hydralazines also showed an association with melanoma.

The reason(s) for our results is(are) unknown. If the results represent true relations, there may exist biologically possible explanations, as it is known that a variety of drugs have affinity for melanin (Larsson, 1993) and that melanocytes contain bioactivating enzymes (Agarwal *et al.*, 1991). Furthermore, many commonly prescribed drugs increase the sensitivity of our skin to sunlight (Hawk, 1990; Allen, 1993). However, it is also possible that the elevated ORs may be due to chance because of the multiple testing in our study. Still another possibility would be that such associations reflect frequency of contact with medical care, and thus diagnostic opportunity. However, oral contraceptive users as well as other prescribed drug users also have contact with the health care system on a regular basis but we found no elevated ORs for melanoma among them. Whether our results on beta-blockers and benzodiazepines may be due to chance, generated by confounding or represent true findings need to be further investigated. In the epidemiological literature almost no attention has been paid to a possible association between pharmaceutical drugs and melanoma. So far, only a few studies have briefly mentioned negative results without displaying data (Beral *et al.*, 1988; Adam *et al.*, 1981; Green *et al.*, 1986).

It has been hypothesised that melanoma development may be promoted by alcohol-induced pituitary secretion of melanocyte-stimulating hormone (Williams, 1976). Several studies have therefore investigated the association between alcohol and melanoma (Williams and Horm, 1977; Green *et al.*, 1986; Holman *et al.*, 1986; Østerlind *et al.*, 1988*a*; Stryker *et al.*, 1990; Adami *et al.*, 1992; Bain *et al.*, 1993; Kirkpatrick *et al.*, 1994). A positive relation has been reported in four studies (William and Horm, 1977; Holman *et al.*, 1986; Stryker *et al.*, 1990; Bain *et al.*, 1993). In a case–control study based on the Third National Cancer Survey, a non-significant positive association between alcohol and melanoma was demonstrated (William and Horm, 1977). Stryker *et al.* (1990) found an alcohol consumption over 10 g per day to be a risk factor for melanoma. Their results for total alcohol intake did not differ between superficial spreading melanoma and other types. Similarly, in a study from Western Australia elevated ORs were found for alcohol consumption in relation to melanoma, albeit significant only for the small group with unclassifiable melanoma (Holman *et al.*, 1986). Moreover, Bain *et al.* (1993) reported that women drinking 20 g or more alcohol per day had 2.5 times the risk of melanoma as non-drinkers. However, the elevated OR was not statistically significant. Except for the latter study none of these positive studies have adequately considered sun exposure as a possible confounder. In contrast, a study by Østerlind et al. (1988a) demonstrated a significant trend for decreasing melanoma risk with increasing alcohol intake. In our study we found no elevated ORs for developing malignant melanoma according to either total alcohol intake or alcohol intake categorised as light beer, beer, wine and distilled liquor. Furthermore, no variations by sex or age were demonstrated. In the present study we did control for sun exposure; however, the results were unchanged. Our findings are in accordance with the results from several other studies (Green et al., 1986; Adami et al., 1992; Kirkpatrick et al., 1994). Therefore, when considering all available data together it seems unlikely that alcohol intake is an independent risk factor for melanoma.

In accordance with previous findings (Williams and Horm, 1977; Paffenberger et al., 1978; Green et al., 1986; Holman et al., 1986; Østerlind et al., 1988a), smoking did not seem to significantly influence melanoma risk.

Since it has been oberved that endogenous female hormones might influence melanoma incidence (Boyle and Robertson, 1987) and prognosis (Holly, 1986), and that hyperpigmentation occurs during pregnancy and oral contraceptive use (Sanchez et al., 1981), it has been hypothesised that there could be an association between sexual hormones (endogenous and/or exogenous) and melanoma development in female. Several studies have therefore, in addition to the results reported on oral contraceptives, to a varying extent also investigated menstrual and reproductive factors (Holly et al., 1983; Holman et al., 1984; Gallagher et al., 1985; Green and Bain, 1985; Østerlind et al., 1988b; Zanetti et al., 1990; Hannaford et al., 1991; Lê et al., 1992). Like Gallagher et al. (1985) we found support for a decrease in melanoma risk (a significant trend) for women with greater number of live births. In an Italian study an inverse association between number of live births and melanoma was found to be diminished by adjustment for education, sun exposure and sun susceptibility (Zanetti et al., 1990). Gallagher et al. (1985) did not take sun exposure into account in their analyses. Six other studies have reported on this issue (Holly et al., 1983; Holman et al., 1984; Green and Bain, 1985; Østerlind et al., 1988b; Hannaford et al., 1991; Lê et al., 1992), and four of them have shown a non-significantly decreased risk (Holly et al., 1983; Holman et al., 1984; Østerlind et al., 1988b; Hannaford et al., 1991). Holly et al. (1983) demonstrated delayed childbearing to be related to increased melanoma risk. This was true only for superficial spreading melanoma and in the analysis only adjustments for oral contraceptive use and education were done. In a recent study menarche before age 14 years was suggested to be associated with melanoma risk (Lê et al., 1992). As have others (Holman et al., 1984; Gallagher et al., 1985; Green and Bain, 1985; Østerlind et al., 1988b), we found no evidence of a significant effect of either age at first birth or age at menarche on melanoma risk. Our findings and the disparity in results between the previous studies suggest no large impact of reproductive and menstrual factors on melanoma risk. However, further studies are needed to draw definitive conclusions on the relation between number of live births and risk of melanoma.

Previous reports of oral contraceptives and malignant melanoma have predominantly failed to shown any association (Bain et al., 1982; Helmrich et al., 1984; Holman et al., 1984; Gallagher et al., 1985; Green and Bain, 1985; Østerlind et al., 1988b; Zanetti et al., 1990). In some studies an increased risk has been observed for long duration of use (Beral et al., 1977, 1984; Adam et al., 1981; Holly et al., 1983; Hannaford et al., 1991; Lê et al., 1992) or for use that started many years before diagnosis and lasted several years (Holly et al., 1983; Beral et al., 1984; Lê et al., 1992). However, most of these estimates were non-siginificant (Beral et al., 1977; Adam et al., 1981; Hannaford et al., 1991) and some estimates were not adjusted for sun exposure variables (Beral et al., 1977; Holly et al., 1983; Hannaford et al., 1991; Lê et al., 1992). In accordance with most studies (Beral et al., 1977; Adam et al., 1981; Bain et al., 1982; Helmrich et al., 1984; Holman et al., 1984; Gallagher et al., 1985; Green and Bain, 1985; Østerlind et al., 1988b; Zanetti et al., 1990; Hannaford et al., 1991; Palmer et al., 1992), our data on duration of oral contraceptive use and time when use took place, do not suggest a significant association between oral contraceptives and melanoma. However, hyperpigmentation during use of oral contraceptives and pregnancy typically occur in the face, but also around the breast and the umbilicus. Only a few studies have performed analyses of risk of site-specific melanoma in relation to oral contraceptive use (Beral et al., 1977, 1984; Zanetti et al., 1990). In one study, with heterogeneous data, an excess of lesions of the lower limbs was reported (Beral et al., 1977). In the present study, as in two other studies (Beral et al., 1984; Zanetti et al., 1990), site-specific analyses (trunk vs extremeties or head and neck) did not show any difference from the overall results. Unfortunately, few, if any, of the studies (including ours) have been large enough to allow site-specific analyses to consider different sites more in detail.

As have others, we found no significant association between hormonal replacement therapy and melanoma risk (Beral et al., 1977, 1984; Adam et al., 1981; Holman et al., 1984; Gallagher et al., 1985; Østerlind et al., 1988b).

To reduce the likelihood of selection bias, it was ascertained that all cases with a first diagnosis of melanoma within a defined area had been included and that controls were randomly selected from the general population of the same area. Furthermore, without knowing our hypothesis, a large percentage of cases and controls answered the comprehensive questionnaire. However, the response rate was somewhat higher for cases than for controls. It is difficult to assess fully the potential bias introduced by the differences in response rate between cases and controls, but there is no *a priori* reason to suspect that identified risk factors in this study are associated with non-participation.

One important methodological issue when performing a multivariate analysis is the issue of which variables to control for (see also above – prescribed drugs). Among possible confounders exposure to sunlight is the most important. The multivariate models included adjustments for number of sunburns as this factor was identified as the most important measure of sun exposure in this case–control study (Westerdahl et al., 1994a). Analyses adjusted for other factors regarding sun exposure gave the same results. However, we cannot exclude that our findings could be due to uncontrolled confounding by a sun exposure variable and/or another variable not measured in the questionnaire.

Another major source of bias in case–control studies is measurement errors. Although the self-administered questionnaire used in this case–control study has been shown to provide information with good test–retest reliability (Westerdahl et al., 1996), the influence of non-differential misclassification may have been substantial, as stated above for prescribed drugs, as measures of exposures analysed were crude. In this context it is important to remember that our data on constitutional factors also relied on self-assessment. However, it is widely appreciated that the effect of non-differential misclassification is to lead to an underestimation of a true relationship. A particular concern in case–control studies is recall bias (i.e. if cases report differently than controls). In the present study identical procedures of data collection for cases and controls were used, the information from cases was collected close in time to the diagnosis in order to avoid the influence that the diagnosis of melanoma may have on recall, and subjects were unaware of our hypothesis. Furthermore, reported smoking habits, which are the object of health concern among the general population, were similar for cases and controls. We therefore do not think that misclassification because of reporting errors differs between cases and controls.

In conclusion, our results suggest that alcohol, smoking, reproductive or hormonal factors do not increase the risk of melanoma. However, increasing number of live births may be protective. Some support is lent to the possibility that specific types of prescribed drugs (beta-blockers, hydralazine and benzodiazepines) may be associated with melanoma. However, these findings were diminished, at least for benzodiazepines, after adjustment for host factors and as they are unconfirmed, and may be due to chance or to confounding, further investigation is needed.

**Acknowledgements**

This study was supported by grants from the Swedish Cancer Society and the Medical Faculty of Lund University.

### References

ADAM SA, SHEAVES JK, WRIGHT NH, MOSSER G, HARRIS RW AND VESSEY MP. (1981). A case–control study of the possible association between oral contraceptives and malignant melanoma. *Br. J. Cancer*, **44,** 45–50.

ADAMI H-O, MCLAUGHLIN JK, HSING AW, WOLK A, EKBOM A, HOLMBERG L AND PERSSON I. (1992). Alcoholism and cancer risk: a population-based cohort study. *Cancer Causes Control*, **3,** 419–425.

AGARWAL R, MEDRANO EE, KHAN IU, NORDLUND JJ AND MUKHTAR H. (1991). Metabolism of benzo(a)pyrene by human melanocytes in culture. *Carcinogenesis*, **12,** 1963–1966.

ALLEN JE. (1993). Drug induced photosensitivity. *Clin. Pharmacol.*, **12,** 580–587.

BAIN C, HENNEKENS CH, SPEIZER FE, ROSNER B, WILLETT W AND BELANGER C. (1982). Oral contraceptive use and malignant melanoma. *J. Natl. Cancer Inst.*, **68,** 537–539.

BAIN C, GREEN A, SISKIND V, ALEXANDER J AND HARVEY P. (1993). Diet and melanoma. An exploratory case–control study. *Ann. Epidemiol.*, **3,** 235–238.

BERAL V, RAMCHARAN S AND FARIS R. (1977). Malignant melanoma and oral contraceptive use among women in California. *Br. J. Cancer*, **36,** 804–809.

BERAL V, EVANS S, SHAW H AND MILTON G. (1984). Oral contraceptive use and malignant melanoma in Australia. *Br. J. Cancer*, **50,** 681–685.

BOYLE P AND ROBERTSON C. (1987). Age-period-cohort modeling of malignant melanoma in Scotland: epidemilogic implications (abstract). *Am. J. Epidemiol.*, **126,** 766.

ELWOOD JM. (1992). Melanoma and sun exposure: contrasts between intermittent and chronic exposure. *World J. Surg.*, **16,** 157–165.

GALLAGHER RP, ELWOOD JM, HILL GB, COLDMAN AJ, THRELFALL WJ AND SPINELLI JJ. (1985). Reproductive factors, oral contraceptives and risk of malignant melanoma: Western Canada Melanoma Study. *Br. J. Cancer*, **52,** 901–907.

GREEN A AND BAIN C. (1985). Hormonal factors and melanoma in women. *Med. J. Aust.*, **142,** 446–448.

GREEN A, BAIN C, MCLENNAN R AND SISKIND V. (1986). Risk factors for cutaneous melanoma in Queensland. *Recent Results Cancer Res.*, **102,** 76–97.

HANNAFORD PC, VILLARD-MACKINTOSH L, VESSEY MP AND KAY CR. (1991). Oral contraceptives and malignant melanoma. *Br. J. Cancer*, **63,** 430–433.

HAWK JLM. (1990). Photosensitivity in the elderly. *Br. J. Dermatol.*, **122,** 29–41.

HELMRICH SP, ROSENBERG L, KAUFMAN DW, MILLER DR, SCHOTTENFELD D, STOLLEEY PD AND SHAPIRO S. (1984). Lack of an elevated risk of malignant melanoma in relation to oral contraceptive use. *J. Natl. Cancer Inst.*, **72,** 617–620.

HOLLY EA. (1986). Melanoma and pregnancy. *Recent Results Cancer Res.*, **102,** 118–126.

HOLLY EA, WEISS NS AND LIFF JM. (1983). Cutaneous melanoma in relation to exogenous hormones and reproductive factors. *J. Natl. Cancer Inst.*, **70,** 827–831.

HOLMAN CDJ, ARMSTRONG BK AND HEENAN PJ. (1984). Cutaneous malignant melanoma in women: exogenous sex hormones and reproductive factors. *Br. J. Cancer*, **50,** 673–680.

HOLMAN CDJ, ARMSTRONG BK, HEENAN PJ, BLACKWELL JB, CUMMING FJ, ENGLISH DR, HOLLAND S, KELSALL GRH, MATZ LR, ROUSE IL, SINGH A, TEN SELDAM REJ, WATT JD AND XU Z. (1986). The causes of malignant melanoma: results from the West Australian Lions Melanoma Research Project. *Recent Results Cancer Res.*, **102,** 18–37.

KIRKPATRICK CS, WHITE E AND LEE JAH. (1994). Case–control study of malignant melanoma in Washington State. II. Diet, alcohol and obesity. *Am. J. Epidemiol,*, **139,** 869–880.

LARSSON BS. (1993). Interaction between chemicals and melanin. *Pigment. Cell Res.,*. **6,** 127–133.

LÊ MG, CABANES PA, DESVIGNES V, CHANTEAU MF, MLIKA N AND AVRIL MF. (1992). Oral contraceptive use and risk of cutaneous malignant melanoma in a case–control study of French women. *Cancer Causes Control*, **3,** 199–205.

ØSTERLIND A, TUCKER MA, STONE BJ AND JENSEN OM. (1988*a*). The Danish case–control study of cutaneous malignant melanoma. IV. No association with nutritional factors, alcohol, smoking or hair dyes. *Int. J. Cancer*, **42,** 825–828.

ØSTERLIND A, TUCKER MA, STONE BJ AND JENSEN OM. (1988*b*). The Danish case–control study of cutaneous malignant melanoma. III. Hormonal and reproductive factors in women. *Int. J. Cancer*, **42,** 821–824.

PAFFENBERGER RS, WING AL AND HYDE RT. (1978). Characteristics in youth predictive of adult-onset malignant lymphomas, melanomas and leukemias: brief communication. *J. Natl. Cancer Inst.*, **60,** 89–92.

PALMER JR, ROSENBERG L, STROM BL, HARLAP S, ZAUBER AG, WARSHAUER ME AND SHAPIRO S. (1992). Oral contraceptive use and risk of cutaneous malignant melanoma. *Cancer Causes Control*, **3,** 547–554.

SANCHEZ NP, PATHAK MA, SATO S, FITZPATRICK TB, SANCHEZ JL AND MIHM MC. (1981). Melasma: a clinical light microscopic ultrastructural and immunofluorescence study. *J. Am. Acad. Dermatol.*, **4,** 698–710.

STRYKER WS, STAMPFER MJ, STEIN EA, KAPLAN L, LOUIS TA, SOBER A AND WILLETT WC. (1990). Diet, plasma levels of beta-carotene and alpha-tocopherol, and risk of malignant melanoma. *Am. J. Epidemiol.*, **131,** 597–611.

WESTERDAHL J, OLSSON H AND INGVAR C. (1994*a*). At what age do sunburn episodes play a crucial role for the development of malignant melanoma. *Eur. J. Cancer.*, **30A,** 1674–1654.

WESTERDAHL J, OLSSON H, MÅSBÄCK A, INGVAR C, JONSSON N, BRANDT L, JÖNSSON P-E AND MÖLLER T. (1994*b*). Use of sunbeds or sunlamps and malignant melanoma in Southern Sweden. *Am. J. Epidemiol.*, **140,** 691–699.

WESTERDAHL J, OLSSON H, MÅSBÄCK A, INGVAR C AND JONSSON N. (1995). Is use of sunscreens a risk factor for malignant melanoma? *Melanoma Res.*, **5,** 59–65.

WESTERDAHL J, ANDERSON H, OLSSON H AND INGVAR C. (1996). Reproducibility of a self-administered questionnaire for assessment of melanoma risk. *Int. J. Epidemiol.* (in press).

WILLIAMS RR. (1976). Breast and thyroid cancer and malignant melanoma promoted by alcohol-induced pituitary secretion of prolactin, T.S.H., M.S.H. *Lancet*, **1,** 996–999.

WILLIAMS RR AND HORM JW. (1977). Association of cancer sites with tobacco and alcohol consumption and socioeconomic status of patients: interview study from the Third National Cancer Survey. *J. Natl. Cancer Inst.*, **58,** 525–547.

ZANETTI R, FRANCESCHI S, ROSSO S, BIDOLI E AND COLONNA S. (1990). Cutaneous malignant melanoma in females: the role of hormonal and reproductive factors. *Int. J. Epidemiol.*, **19,** 522–526.